# Association between occupational balance and the physical and mental health of the university community: an observational study

**DOI:** 10.3389/fpubh.2025.1631096

**Published:** 2025-07-31

**Authors:** Elisabet Huertas-Hoyas, Cristina García-Bravo, Jorge Pérez-Corrales, Elisa Bullón-Benito, Gemma Fernández-Gómez, Mª Pilar Rodríguez-Pérez

**Affiliations:** ^1^Department of Physical Therapy, Occupational Therapy, Rehabilitation and Physical Medicine, Rey Juan Carlos University, Madrid, Spain; ^2^Research Group of Participation, Roles, Occupations and Activities for Community Transformation (PROACT), Alcorcón, Spain; ^3^Research Group of Humanities and Qualitative Research in Health Science (Hum&QRinHS), Alcorcón, Spain; ^4^Center for Comprehensive Care for Children and Adolescents (TANGRAM), Madrid, Spain

**Keywords:** university students, occupational balance, mental health, physical health, work

## Abstract

**Aims:**

The study aims to explore the influence of occupational balance on the physical and mental health of working university students and to describe the existing correlations within the university community between sociodemographic variables and physical and mental health.

**Materials and methods:**

This is a descriptive cross-sectional study. Participants were recruited through a convenience sampling strategy, targeting students enrolled at the university who met the inclusion criteria and voluntarily agreed to participate. The assessment instruments used were: Occupational Balance Questionnaire, Spanish version (OBQ-E), Depression, Anxiety and Stress Scale 21 (DASS-21), and the Spanish version of the International Physical Activity Questionnaire Short Form (IPAQ-SF).

**Results:**

A sample of 89 participants was obtained, with a mean age of 25.42 ± 8.33. Significant differences were observed in sociodemographic and emotional variables between working and non-working students. The results indicate that non-working students have higher levels of anxiety (*t* = 2.7, *p* < 0.01), while working students show lower levels of occupational balance (*t* = 0.195, *p* < 0.05) and prefer light physical activities over moderate ones. Additionally, significant negative correlations were found between age and anxiety (*r* = −0.440, *p* < 0.0001), depression (*r* = −0.238; *p* < 0.05), stress (*r* = −0.399, *p* < 0.001) and vigorous activity (*r* = −0.223, *p* < 0.05), as well as between occupational balance and anxiety (*r* = −0.334, *p* < 0.0001), depression (*r* = −0.443, *p* < 0.0001), and, vigorous activity (*r* = +0.283, *p* < 0.001) stress (*r* = −0.531, *p* < 0.0001) variables.

**Conclusion:**

Occupational balance has a significant impact on the physical and mental health of working university students. Those with better occupational balance show lower levels of depression, anxiety, and stress. Further studies with larger samples are needed to corroborate these findings.

## 1 Introduction

Physical activity is scarce for many university students (1). Additionally, with the increasing use of smartphones, playing computer games, and using interactive movie and series platforms, the time spent sitting is constantly increasing compared to the time dedicated to physical activities (2).

Technological advances have increased comfort; however, they have also led to a decrease in physical activity, resulting in an increase in the incidence of various diseases. According to the World Health Organization, more than 60% of the world’s population does not engage in moderate physical activity (30 min per day) (3). Engaging in physical activity has long been associated with positive outcomes for physical and mental health. However, the recent increase in sedentary behaviors due to work, computer games, smartphones, and television platforms deserves attention from a healthcare management perspective (1). University students often spend extended periods sitting during lectures, studying, and using computers or smartphones, which can lead to negative consequences such as musculoskeletal discomfort, increased stress levels, and reduced physical activity. Furthermore, prolonged sedentary behavior in this population has been associated with a higher risk of developing poor physical and mental health outcomes, including weight gain, anxiety, and depression, which may also impact academic performance.

Sedentary behaviors are those that involve lower energy expenditure during waking hours and include both sitting and lying down. Only a limited number of studies have measured the time university students spend sitting. In a study conducted with Polish students (4), it was indicated that physical activity decreases with age, and differences in exercise intensity and type by gender were observed. Additionally, most studies have focused on adults. A study conducted by Bauman et al. (5) monitored the time adults from 20 countries spent sitting, finding a daily average of 5.8 h. The authors reported that adults in the United States, Canada, and the United Kingdom remained seated between 9 and 11 h a day, which represented between 55 and 70% of their waking hours. However, the scoping review conducted by McLaughlin et al. (6) revealed significant differences between countries, with the time citizens spent sitting varying from 2.2 to 9.5 h daily. This review observed that citizens of high-income countries spent nearly twice as much time sitting compared to those in low-income countries, with averages of 4.9 and 2.7 h per day, respectively.

Maintaining a lifestyle centered around sitting can pose health risks in university students. Recent studies (7–9) confirm that individuals with high levels of sedentary behavior have an increased risk of obesity, type 2 diabetes, loss of bone density, cardiovascular disorders, and certain types of cancer compared to those who spend less time sitting.

In studies conducted among university students, regular physical activity and reduction in sedentary behavior have been associated with better mental health (10–12). The study by Reyes-Molina et al. (11) among Chilean university students showed that high levels of sedentary behavior were associated with lower subjective well-being and worse mental health, while staying physically active improved these indicators. Additionally, various studies (10, 12) indicate that replacing sedentary time with regular physical activity significantly reduces symptoms of depression and anxiety, as well as improves stress management and academic performance, which is related to better mental health (13).

In a study conducted with university students in Spain, it was determined that students who spent more than 42 h a week sitting had a 31% higher risk of developing mental disorders compared to students who did not (14). Nevertheless, although current research suggests that physical activity, mental health, and well-being are positively related in university students (15), there is a scarcity of studies showing the relationship between occupational balance and physical and mental health. Occupational balance is a fundamental concept in occupational therapy, defined as individuals’ perception and satisfaction with the quality and quantity of the occupations they engage in daily such as work, leisure, self-care, and social participation; a lack of occupational balance, or occupational imbalance, occurs when there is an overemphasis on certain activities to the detriment of others, leading to potential negative outcomes for health and well-being (16). The study by Guszkowska and Dąbrowska-Zimakowska (17) conducted among Polish university students during the COVID-19 pandemic showed that changes in occupational balance, with increased time spent on the internet and passive rest, negatively affected their psychological well-being. Excessive focus on academic tasks and prolonged sedentary behaviors can lead to stress, anxiety, and musculoskeletal issues, while insufficient engagement in leisure and social activities may exacerbate feelings of isolation or burnout (17). Conversely, maintaining occupational balance could promote psychological resilience, physical activity, and overall well-being. In the Spanish context, Romero-Tébar et al. (18) showed that university students with better occupational balance exhibited lower problematic internet use. Recent studies have demonstrated that an imbalance in daily activities can contribute to sedentary behavior and, consequently, to metabolic, cardiovascular and musculoskeletal diseases. Qualitative research has highlighted the importance of occupational balance in improving well-being, but few studies have quantitatively addressed its effects on physical health (19).

The main objective of this study is to examine the relationship between occupational balance and both physical and mental health within the university community, including both working and non-working individuals, with students comprising the majority of participants. Additionally, the study seeks to examine the correlations between sociodemographic variables (e.g., age, gender, role, working, etc.) and physical and mental health in the university population; and to identify potential differences in levels of occupational imbalance based on specific sociodemographic variables.

## 2 Materials and methods

### 2.1 Design

A cross-sectional, descriptive design was used, following the guidelines of the Strengthening the Reporting of Observational Studies in Epidemiology (STROBE) checklist (20).

This study was approved by the Rey Juan Carlos University Research Ethics Committee, reference number 2802202310523. All participants were informed in advance regarding the procedures, risks, and benefits of the research. In addition, in-formed consent forms were requested to be signed and provided at the start of the study, in accordance with the Declaration of Helsinki, as revised at the World Medical Association Assembly in 2013 (21).

### 2.2 Participants

The study was conducted at Rey Juan Carlos University, Madrid, Spain. The inclusion criteria were being over 18 years old and being a member of the university community (undergraduate, master’s, and doctoral students, as well as teaching and research staff (teachers), and technical, administrative and support staff). The exclusion criteria were having any limitation or restriction in physical mobility that impedes walking and static standing (only for those cases where physical assessment would be conducted, due to the conditions of the test). All participants were required to agree to participate and sign an informed consent form in accordance with the most recent version of the Declaration of Helsinki (21).

The proposed sample size was estimated using the G*Power program (22) based on previous research findings that reported correlations ranging from 0.31 to 0.72 between occupational balance and physical and mental health in adults (23). Therefore, a minimum of 76 participants was required to estimate correlations of at least 0.31 with 95% confidence and 80% statistical power. A minimum of 76 individuals from the university community were recruited. Participants were recruited through social media platforms (e.g., Twitter and Instagram) and official university communication channels (e.g., emails, website announcements). Invitations to participate were extended to all members of the university community, including students, teaching and research staff, and technical, administrative and support staff.

### 2.3 Data collection

Data were collected online using a Microsoft Forms questionnaire (Microsoft Office 365 application) regarding physical and mental health status, as well as sociodemographic data. Physical health will be assessed using indicators such as sleep quality, physical activity levels, and the presence of self-reported chronic conditions or illnesses; mental health will be measured through validated questionnaires evaluating perceived stress levels, symptoms of anxiety and depression, and overall life satisfaction; occupational imbalance will be addressed using an instrument that evaluates students’ perceptions of the distribution and management of their time across meaningful activities (academic, recreational, and social).

The estimated time to complete the questionnaire is 20 min. The following are the assessment instruments used to gather the information:

- Sociodemographic: Data will be collected on gender, age, type of studies, place of residence, and employment status; if employed, the number of hours worked per week.
- Occupational Balance Questionnaire (OBQ): This is a questionnaire in which individuals assess their occupational balance in relation to their daily life situation. It consists of 13 items exploring occupational balance, meaning, time use, and satisfaction. Responses are rated on a six-point scale, with values ranging from 0, “Strongly Disagree,” to 5, “Strongly Agree.” A total score can range from 0 to 65, with higher scores indicating greater occupational balance. The OBQ has been adapted and validated for the Spanish population (OBQ-E), demonstrating good psychometric properties (24).
- Depression, Anxiety, and Stress Scale 21 (DASS-21): This is a brief and easy-to-complete self-report instrument consisting of 21 items divided into three subscales of 7 items each. Each item is rated on a Likert scale from 0 to 3 points, with higher scores indicating greater symptoms of depression, anxiety, and stress. The Spanish version has demonstrated good psychometric properties in terms of internal consistency, convergent validity, and divergent validity (25).
- International Physical Activity Questionnaire (IPAQ): The level of physical activity in the sample will be measured using the Spanish version of the International Physical Activity Questionnaire Short Form (IPAQ-C). This is a self-administered questionnaire consisting of 7 items that inquire about the physical activities performed by the individual over the past 7 days. The final score, expressed in MET-minutes per week, is derived from the number of days, minutes, and hours spent on physical activities, and allows classification of the individual as sedentary (<600 MET-minutes/week), moderately active (600–1,500 MET-minutes/week), or highly active (>1,500 MET-minutes/week). The psychometric properties of the IPAQ-C support its use in population-based physical activity studies (reliability of 0.65) (26).

### 2.4 Data analysis

Statistical analyses were performed using IBM SPSS Statistics for Windows, version 27.0 (IBM SPSS Corp., Armonk, NY, United States).

First, a descriptive analysis of all variables of interest was conducted. Quantitative variables (such as age, occupational balance, or internalizing symptoms) were expressed using means and standard deviations, and qualitative variables (such as gender, physical activity level, or university community group) were described using absolute and relative frequencies.

Second, a bivariate analysis was carried out between the main dependent variable (occupational balance) and the other variables of interest. This was involve checking the distribution of quantitative variables using the Kolmogorov–Smirnov test. The Kolmogorov–Smirnov test was used to assess the normality of the distribution of continuous variables included in the study. This test was chosen due to the anticipated sample size (>30), as it is suitable for detecting significant deviations from normality in moderate to large samples. Different hypothesis testing strategies were employed: the association between two quantitative variables was analyzed using Pearson or Spearman correlation coefficients, as appropriate. Differences in means between two groups were assessed using Student’s *t*-test for independent samples or the Mann–Whitney U test, as appropriate. Finally, the relationship between the presence or absence of occupational imbalance and qualitative variables was examined using the Chi-square test and odds ratios.

Third, multivariate regression analyses were performed to determine the independent variables that contribute to explaining occupational balance both quantitatively (using linear regression analysis) and qualitatively (using binary logistic regression analysis). In all cases, the level of statistical significance was set at *p* < 0.05.

## 3 Results

Table 1 presents the demographic data for a sample of 89 participants. The mean age was 25.42 ± 8.33 years, with 72.3% identifying as female and 15.8% as male. After checking the normality of the sample, parametric tests were used.

**Table 1:** Descriptive basal parameters.

	Overall (*n* = 89)	Working (*n* = 45)	No working (*n* = 44)	t/X^2^	*d*
Age (min-max/M ± SD)	19–59/25.42 ± 8.33	20–59/29 ± 10.47	19–26/21.7 ± 1.54	−4.5***	0.38
Sex				0.25	
Male (Fr (%))	16 (15.8)	9 (20)	7 (15.9)		
Female (Fr (%))	73 (72.3)	36 (80)	37 (84.1)		
Role				20.5***	
Student (Fr (%))	72 (71.3)	28 (62.2)	44 (100)		
TRS (Fr (%))	15 (14.9)	15 (33.3)	0		
TASS (Fr (%))	2 (2)	2 (4.4)	0		
Year				2.66	
1°–3° (Fr (%))	36 (35.7)	12 (26.7)	24 (54.6)		
4°–5° (Fr (%))	33 (32.7)	14 (31.1)	19 (43.2)		
Residence				5.21*	
Inside (Fr (%))	75 (74.3)	34 (75.6)	41 (93.2)		
Outside (Fr (%))	14 (13.9)	11 (24.4)	3 (6.8)		
Hours W (min-max/M ± SD)	2–60/23.43 ± 15.69	2–60/23.43 ± 15.69	–		
OBQ-E (min-max/M ± SD)	25–78/50.85 ± 11.26	34–78/50.6 ± 12.5	25–68/51.09 ± 9.86	0.195*	11.33
DASS_depr (min-max/M ± SD)	0–20/5.17 ± 4.78	0–17/4.29 ± 4.33	0–20/6.07 ± 5.09	1.04	4.93
DASS_anx (min-max/M ± SD)	0–20/5.6 ± 5.1	0–17/4.2 ± 4.04	0–20/7.02 ± 5.69	2.7**	5.06
DASS stress (min-max/M ± SD)	0–20/9.25 ± 5.2	0–18/7.96 ± 4.71	0–20/10.5 ± 5.38	1.35	5.06
IPAC_Vigo (min-max/M ± SD)	0–16,800/1711.6 ± 2476.5	0–16,800/1644.1 ± 2760.6	0–10,080/1780.7 ± 2177.9	0.69	2489.77
IPAC_Moderate (min-max/M ± SD)	0–40,000/1362.8 ± 5480.7	0–10,080/772.6 ± 1593.09	0–40,000/1967.1 ± 7624.9	1.02*	5478.95
IPAC_walking (min-max/M ± SD)	0–11,088/11086.8 ± 1501.5	0–11,088/1355.1 ± 1954.2	66–4,158/1014.7 ± 803.9	1.07*	1500.31
IPAC_total (min-max/M ± SD)	0–42,986/4261.3 ± 6692.6	0–33,810/3771.2 ± 5204.9	99–42,986/4762.6 ± 7964.7	1.21	6712.35

*p < 0.05; **p < 0.01; ***p < 0.001. M, mean; DS, Standard Deviation; TRS, Teaching and Research Staff; TASS, Technical, Administrative and Support Staff; Hours W, hours worked; Dass_depr, Dass depression; Dass_anx, Dass anxiety; IPAC_vigo, IPAC vigorous.

A comparative analysis between working and non-working individuals within the university community revealed differences in several sociodemographic variables, such as age, role, and residence (living with or away from parents). Additionally, differences were observed in variables such as occupational balance (*t* = 0.195, *p* < 0.05), measured by the OBQ-E, with lower scores in the working group; and anxiety (*t* = 2.7, *p* < 0.01), measured by the DASS-21, with higher values in the non-working group, indicating higher anxiety symptoms among non-working individuals. Regarding physical activities, significant differences were found in moderate physical activities, with higher levels in the non-working group (*t* = 1.02, *p* < 0.05), and in light activities (walking), with the working group showing lower levels (*t* = 1.07, *p* < 0.05).

Correlation analysis across the entire sample (Table 2) shows significant negative correlations between personal variables such as age and levels of anxiety or depression, indicating that younger age is associated with higher anxiety (rho = −0.357; *p* < 0.001) and depression (rho = −0.238; *p* < 0.05). Regarding physical activity, a negative correlation with age was observed, showing that as age increases, vigorous activity decreases (rho = −0.223; *p* < 0.05). Significant negative correlations were also found between occupational balance and the variables of depression, anxiety, and stress measured by the DASS-21, indicating that higher occupational balance scores are associated with lower levels of these variables (rho = −0.443; *p* < 0.001; rho = −0.334; *p* < 0.001; rho = −0.531; *p* < 0.001, respectively). Among the emotional variables (anxiety, depression, and stress) measured by the DASS-21, anxiety showed the most significant correlations, including with gender (rho = −0.228; *p* < 0.05), age (rho = −0.357; *p* < 0.001), employment status (rho = −0.278; *p* < 0.01), role (rho = −0.357; *p* < 0.001), occupational balance (rho = −0.334; *p* < 0.001), depression (rho = 0.753; *p* < 0.001), and stress (rho = 0.767; *p* < 0.001).

**Table 2:** Correlations between variables (*n* = 89).

	Sex	Age	Work	Hours W	Role	OBQ-E	Dass_depr	Dass_anx	Dass_stres	IPAC_vigo	PAC_moderate	IPAC_walking	IPAC_total
Sex	1	0.022	0.053	0.369*	0.227*	0.265*	−0.214*	−0.228*	−0.192	0.256*	−0.063	−0.012	0.041
Age	0.022	1	0.440***	0.604**	0.805***	0.071	−0.238*	−0.357***	−0.399***	−0.223*	−0.067	−0.145	−0.170
Work	0.053	0.440***	1	**–**	0.458***	−0.021	−0.187	−0.278**	−0.253*	−0.028	−0.110	0.114	−0.074
Hours W	0.369*	0.604**	–	**1**	0.740**	0.103	−0.279	−0.343*	−0.260	−0.188	−0.214	−0.212	−0.245
Role	0.227*	0.805***	0.458***	0.740**	1	0.152	−0.329**	−0.357***	−0.380***	−0.177	−0.070	−0.079	−0.141
OBQ-E	0.265*	0.071	−0.021	0.103	0.152	1	−0.443***	−0.334***	−0.531***	0.283**	0.039	−0.027	0.130
Dass_depr	−0.214*	−0.238*	−0.187	−0.279	−0.329**	−0.443***	1	0.753***	0.728***	−0.128	0.005	−0.059	−0.056
Dass_anx	−0.228*	−0.357***	−0.278**	−0.343*	−0.357***	−0.334***	0.753***	1	0.767***	−0.095	−0.023	0.155	−0.019
Dass_stress	−0.192	−0.399***	−0.253*	−0.260	−0.380**	-0.531***	0.728***	0.767***	1	−0.029	−0.046	0.096	−0.027
IPAC_vigo	0.256*	−0.223*	−0.028	−0.188	−0.177	0.283**	−0.128	−0.095	−0.029	1	0.135	0.265*	0.540***
IPAC_moderate	−0.063	−0.067	−0.110	−0.214	−0.070	0.039	0.005	−0.023	−0.046	0.135	1	0.044	0.879***
IPAC_walking	−0.012	−0.145	0.114	−0.212	−0.079	−0.027	−0.059	0.155	0.096	0.265*	0.044	1	0.358***
IPAC_total	0.041	−0.170	−0.074	−0.245	−0.141	0.130	−0.056	−0.019	−0.027	0.540***	0.879***	0.358***	1

*The correlation is significant at *p* < 0.05; **The correlation is significant at *p* < 0.01; ***The correlation is significant at *p* < 0.001 Hours W, hours worked; Dass_depr, Dass depression; Dass_anx, Dass anxiety; IPAC_vigo, IPAC vigorous. Data in bold showed statistically significant correlations.

## 4 Discussion

The aim of this study was to explore the influence of occupational balance on the physical and mental health of university students. Additionally, it sought to describe the existing correlations between sociodemographic variables and physical and mental health within the university community.

According to the findings, significant differences exist between university students who work and those who are not employed in terms of occupational balance, age, role, place of residence, anxiety, and both moderate and light physical activity. It appears that working individuals show better values in terms of emotional symptoms; however, in terms of physical activity, they are characterized by engaging in light rather than vigorous or moderate exercise. Different studies (27, 28) have reflected that university students who work may experience benefits in their emotional health compared to those who do not. According to these studies, balancing employment with studies can provide a sense of purpose and achievement, improve time management skills, and reduce financial stress, all of which contribute positively to emotional health. In the systematic review conducted by Campbell et al. (27) it was found that students who work show lower levels of anxiety and depression, attributed to the structure and social support provided by the work environment. Regarding physical health, the effects of work can be more varied. Some studies indicate that working students tend to engage in lower levels of vigorous or moderate physical activity, preferring instead light physical activities. This may be due to a lack of time and energy after work and academic obligations (29). Research indicates that students who work part-time are less likely to meet the recommendations for vigorous physical activity (VPA) compared to their non-working peers. The study conducted by Wilson et al. (28) found that part-time employment was associated with a 14% lower likelihood of meeting aerobic activity guidelines and a 31% lower likelihood of meeting muscle-strengthening guidelines overall.

The recent meta-analysis conducted by Yuan et al. (30) indicated that, while university students generally engage in adequate levels of light physical activity, they often fail to meet the recommended levels of moderate or vigorous physical activity, which is essential for maintaining good cardiovascular and muscular health. This research also highlighted the importance of identifying and describing students’ activity patterns to design effective interventions that promote healthy habits and routines. Although working university students may benefit emotionally from their employment, it is crucial for them to find ways to incorporate moderate or vigorous physical activity into their routines to ensure their physical well-being and achieve adequate occupational balance. This is essential to maximize benefits and mitigate potential negative effects of work, as this imbalance can negatively impact their overall health and well-being (31).

Regarding the analysis of relationships between variables, the data reveal significant negative correlations between occupational balance and depression, anxiety and stress. Higher scores in occupational balance are associated with lower levels of these variables. These findings align with existing research, such as the study (32) which found that younger generations, such as Millennials and Generation Z, experience higher levels of stress and anxiety. It appears that younger individuals may face greater levels of anxiety due to uncertainty about the future, academic and work pressures, and less effective coping strategies compared to older adults. Additionally, hormonal and neurological changes during puberty may increase emotional reactivity and susceptibility to stress and anxiety. A study by Narmandakh et al. (33) also showed that biological, psychological, and social changes contribute to anxiety in younger populations. More longitudinal studies are needed to fully understand the dynamics of anxiety in these groups.

Regarding activity levels, a negative correlation with age was observed, indicating that as age increases, vigorous activity decreases. A study published in the Journal of American College Health (34) found that older students have less free time due to their multiple commitments, which contributes to a decrease in levels of vigorous physical activity. This increase in responsibilities may lead to a reduction in the time available for vigorous physical activity.

In this vein, the study by Sáez et al. (35) indicated that older students exhibited lower levels of vigorous physical activity. The study highlights that factors such as increasing academic and work responsibilities with age can influence the decline in physical activity. Similarly, authors like Kljajević et al. (31) have noted that physical capacity and health may be impacted by age, making vigorous activities more difficult to sustain. This study concluded that older university students report more health problems and fatigue, contributing to a decrease in vigorous physical activity.

Our study contributes to providing a solid foundation regarding the negative correlation between age and vigorous physical activity levels among university students. The literature suggests that as students age, they engage less in intense physical activities, which may be related to various factors, including changes in priorities and intrinsic motivation (28, 35). Therefore, this highlights the need for effective interventions focused on motivation and enhancing students’ self-efficacy.

According to the data found, it appears that anxiety is the emotional variable with the most significant relationships, including with sex, age, employment, role, occupational balance, depression, and stress. In our study, there are more women than men, which aligns with the trend observed in healthcare professions, where women often outnumber men. However, in a study conducted by Ahmed et al. (36) found that anxiety tended to be more prevalent and severe among women compared to men. This suggests a significant relationship between sex and levels of anxiety. On the other hand, as previously discussed, age appears to significantly influence anxiety management. The review conducted by Campbell et al. (27) identified that the transition to university and the initial years of study are critical periods for the emergence of mental health issues. Younger students tend to exhibit higher levels of anxiety and stress, particularly those in their early years of university. Transitions and adjustment to university life can be significant stressors for younger students. On the other hand, a study (37), observed that as students experience a poorer work-life balance, they report higher levels of anxiety and depressive symptoms, highlighting the relationship between workload and anxiety. This suggests that the surrounding context can be crucial for effective anxiety management. As indicated by the study of Garett et al. (38) in which they found that students who must fulfill multiple roles, such as being workers or parents in addition to being students, exhibit higher levels of anxiety due to the numerous responsibilities and lack of time. These conditions can also contribute to an occupational imbalance, leading to increased stress and anxiety. Several studies (39–41) have determined that students who manage their time poorly experience an occupational imbalance between their academic responsibilities and leisure time, resulting in high levels of anxiety. Additionally, occupational balance may reduce anxiety not only by improving time management skills but also by providing a sense of control over daily activities. A better balance between academic, work, and personal responsibilities can improve overall well-being, reducing cognitive and emotional overload that contributes to stress and anxiety. Given this situation, if university students exhibit high levels of anxiety, according to our findings, this may be related to unbalanced levels of depression and stress, as indicated by the study of Ibrahim et al. (42). Finally, a recent study Neufeld-Kroszynski et al. (43), observed that academic stress is a significant predictor of anxiety among university students. The study highlights that stress related to studies, exams, and academic pressure significantly contributes to anxiety levels in students. Those with higher levels of academic stress also exhibited higher levels of anxiety, which negatively impacted their academic performance and overall well-being.

This study has limitations. Firstly, the sample size may not be representative of the broader population of university students, which limits the ability to generalize the results. However, the smaller sample size has allowed for more detailed and we analyzed the data provided by each participant within the context of a statistical analysis. Providing a deeper understanding of their experiences and underlying dynamics. Another limitation is that the sample did not include sufficient diversity in key variables such as age and sex, which could introduce bias into the results. Therefore, future studies with larger and more diverse samples are needed to validate these findings and provide a more robust and generalizable understanding of the impact of work on university students’ health.

One of the main limitations of this study is the cross-sectional design employed, which, by its nature, does not allow causal relationships between the analyzed variables to be established. This means it is not possible to determine the directionality of the observed associations, limiting our ability to understand whether occupational balance directly impacts physical and mental health or if these dimensions influence occupational balance. To address this limitation, future studies could employ a longitudinal design, which would allow for the analysis of temporal dynamics and exploration of potential causal relationships between these variables. This approach would provide a more robust and evidence-based understanding of the links between occupational balance and health in the university context. Also, another limitation of this study is the lack of detailed information regarding the characteristics of the students’ employment. Variables such as number of working hours, whether the job is essential for financial support or serves as supplementary income, and whether it is related to the student’s field of study were not collected. These aspects could significantly influence occupational balance and mental health outcomes. Therefore, caution is needed when interpreting the observed differences in anxiety levels between working and non-working students, as unmeasured employment-related factors may have biased the results.

## 5 Conclusion

This research has demonstrated that occupational balance significantly impacts the physical and mental health of working university students. Those with better occupational balance exhibit lower levels of depression, anxiety, and stress, contributing to their overall well-being and health. Specifically, it is linked to improved physical health, such as better engagement in physical activity and overall fitness. This highlights the importance of implementing effective university programs that assist in managing time between work, study, and leisure.

Despite the significant findings, further longitudinal studies and subgroup analyses are needed to fully understand the underlying dynamics between work, occupational balance, and the physical and emotional health of university students. This will aid in developing more effective intervention strategies to enhance the well-being of this population. For instance, interventions such as targeted time management workshops, stress management programs, and physical activity promotion could be valuable in helping students achieve a better balance and improve their overall health and well-being.

## Data Availability

The raw data supporting the conclusions of this article will be made available by the authors, without undue reservation.
