# Correction: Association between occupational balance and the physical and mental health of the university community: an observational study

**DOI:** 10.3389/fpubh.2025.1680962

**Published:** 2025-08-22

**Authors:** Frontiers Production Office

**Affiliations:** Frontiers Media SA, Lausanne, Switzerland

**Keywords:** university students, occupational balance, mental health, physical health, work

The affiliations were erroneously numbered and assigned as follows:

Elisabet Huertas-Hoyas^1, 2†^, Cristina García-Bravo^1, 2, 3†^, Jorge Pérez-Corrales^1, 3†^, Elisa Bullón-Benito^1*†^, Gemma Fernández-Gómez^3, 4†^ and Mª Pilar Rodríguez-Pérez^1, 2†^

^1^Department of Physical Therapy, Occupational Therapy, Rehabilitation and Physical Medicine, Rey Juan Carlos University, Madrid, Spain

^2^Center for Comprehensive Care for Children and Adolescents (TANGRAM), Madrid, Spain

^3^Research Group of Participation, Roles, Occupations and Activities for Community Transformation (PROACT), Alcorcón, Spain

^4^Research Group of Humanities and Qualitative Research in Health Science (Hum&QRinHS), Alcorcón, Spain

The affiliations have now been renumbered and assigned as follows:

Elisabet Huertas-Hoyas^1, 2†^, Cristina García-Bravo^1, 2, 3†^, Jorge Pérez-Corrales^1, 3†^, Elisa Bullón-Benito^1*†^, Gemma Fernández-Gómez^2, 4†^ and Mª Pilar Rodríguez-Pérez^1, 2†^

^1^Department of Physical Therapy, Occupational Therapy, Rehabilitation and Physical Medicine, Rey Juan Carlos University, Madrid, Spain

^2^Research Group of Participation, Roles, Occupations and Activities for Community Transformation (PROACT), Alcorcón, Spain

^3^Research Group of Humanities and Qualitative Research in Health Science (Hum&QRinHS), Alcorcón, Spain

^4^Center for Comprehensive Care for Children and Adolescents (TANGRAM), Madrid, Spain

The original version of this article has been updated.

